# Metanephric adenoma with BRAF V600K mutation and a doubtful radiological imaging: pitfalls in the diagnostic process

**DOI:** 10.1007/s00795-020-00269-z

**Published:** 2020-11-11

**Authors:** Niccolo Lenci, Pierconti Francesco, Eros Scarciglia, Vincenzo Fiorentino, Mattia Schino, Giuseppe Palermo, Marco Racioppi, Pierfrancesco Bassi, Maurizio Martini

**Affiliations:** 1 Fondazione Policlinico Universitario A. Gemelli IRCCS, Università Cattolica del Sacro Cuore, Largo A. Gemelli 8, 00168 Rome, Italy; 2 Divisione di Anatomia Patologica, Dipartimento di scienze della vita e sanità pubblica, Università Cattolica del Sacro Cuore A. Gemelli, Rome, Italy; 3 Divisione di Urologia, Dipartimento di medicina e chirurgia traslazionale, Università Cattolica del Sacro Cuore A. Gemelli, Rome, Italy

**Keywords:** Metanephric adenoma, BRAF V600K, BRAF VE1 antibody

## Abstract

Metanephric adenoma (MA) is an uncommon benign renal tumor whose histomorphological aspect resembles that of Wilms’ tumor and papillary renal cell carcinoma. From a diagnostic and therapeutic perspective, recognition of this entity is important as it has a more favorable clinical outcome compared with Wilms’ tumor and papillary renal cell carcinoma. MA should not be treated with nephrectomy if the tumor size is small, opting for a conservative treatment. However, the preoperative diagnosis of this disease is extremely challenging. The present study describes a case of this rare disease, showing an ambiguous radiological imaging and that only after a percutaneous biopsy, was defined as a MA and treated with partial nephrectomy. Moreover, the histological diagnosis of this case was partially complicated by the equivocal immunohistochemical analysis showing negativity for BRAF VE1 staining. Only the mutational analysis demonstrated the presence of the BRAF V600K mutation (for the first time described in a case of metanephric adenoma), highlighting the necessity of sequencing in case of MA with negativity for BRAF VE1 clone.

## Introduction

Metanephric adenoma (MA) is a rare tumor, accounting for 0.2% of adult renal epithelial neoplasms [1]. It occurs most commonly in middle-aged women and is considered benign [2]. Radiological diagnosis of this benign tumor is typically difficult, and most MAs are misdiagnosed as renal cell carcinomas (RCCs) preoperatively. However, accurate diagnosis is of great importance as it may avoid unnecessary radical surgery, especially if the lesion has a small size [1, 3]. From a histological perspective, metanephric adenoma should be differentiated from Wilms' tumor, oncocytoma and papillary RCC. Recently studies demonstrated that about 80–90% of MA have a BRAF V600E mutation, making this molecular alteration and the use of BRAF VE1 antibody helpful in the differential diagnosis [4]. In the present report, we describe a clinical case along with its immunohistochemical, molecular and radiological findings, highlighting the pitfalls and the helpful analytical procedures in the diagnostic process.

## Materials and methods

### Immunohistochemial analysis

We performed immunostaining on formalin-fixed, paraffin-embedded (FFPE) sections of the primary tumor using the antibodies listed in Table 1, following the condition indicated in Table 1 and a OMNIS (DakoCytomation, Carpinteria, CA, USA) or VENTANA BenchMark ULTRA automatic immunostainer (Roche Tissue Diagnosis, Oro Valley, AZ, USA). We used Image J (National Institutes of Health, Bethesda, MD, USA) to estimate the percentage of Ki-67-positive tumor cells. The results of the immunohistochemical examinations are summarized in Table 1.

**Table 1:** The antibody used in this study and their results

Antigen	Clone	Source	Equip	Ant-R	MA
CK AE1/AE3	AE1/AE3	DakoCytomation	D	pH6 (10 min.)	+ + +
WT1	6F-H2	DakoCytomation	D	pH8 (20 min.)	+ + +
CD57	TB01	DakoCytomation	D	pH8 (20 min.)	+ +
PAX8	MRQ-50	RocheVentana	R	pH8 (20 min.)	+ +
CK7	OUTL 12/30	DakoCytomation	D	pH8 (20 min.)	+ (focal)
VIMENTIN	V9	DakoCytomation	D	pH6 (10 min.)	+ (focal)
CD10	SP67	RocheVentana	R	pH8 (20 min.)	−
P504S	13H4	Leica bisystems	L	pH8 (20 min.)	−
BRAF VE1	VE1	RocheVentana	R	pH8 (20 min.)	−
CK20	KS20.8	DakoCytomation	D	pH8 (20 min.)	−
Ki-67	39–9	RocheVentana	R	pH8 (20 min.)	2–3%

*Equip* equipment, *Ant-R* antigen retrieval, *MA* metanephric adenoma, *CK* cytokeratin, *D* Dako Omnis, *R* Roche VENTANA Benchmark ULTRA automatic immunostainer, − negative, f + focally positive (1–20%), +  + positive (20–50%), +  +  + diffusely positive (> 51%)

### Genetical analysis

According to the previously described method [5], BRAF gene mutation was examined. Briefly, DNA was extracted from an FFPE sample of the primary tumor using the QIAamp DNA FFPE tissue kit (Qiagen, Hilden, Germany). The polymerase chain reaction (PCR) was performed with the Go Taq DNA polymerase kit (Promega, Milan, Italy). The PCR products were electrophoresed, and each purified product was directly sequenced using BRAF forward and reverse primers with the BigDye Terminator v3.1 cycle sequencing kit (Thermo Fisher Scientific, Waltham, MA, USA) using an ABI PRISM 3500 Genetic Analyzer (Thermo Fisher Scientific, Waltham, MA, USA).

## Results

### Clinical history

**Fig. 1:**
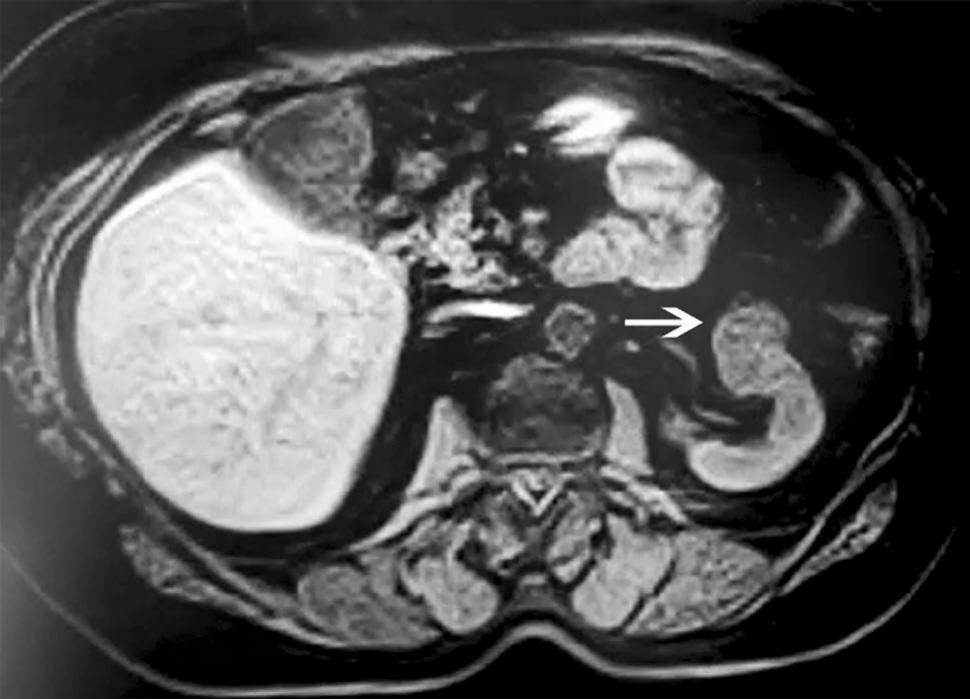
The figure shows the RMI imaging of a lesion (metanephric adenoma; white arrow) of 3 cm situated in the middle of the left kidney

A non-smoker 73-year-old patient with two previous pregnancies, presented at our Urological Department in March 2020 following the incidental ultrasound examination of a left renal neoformation. The patient had no pain or palpable abdominal masses and denied previous episodes of macrohematuria. Blood test and chest X-Ray were normal. The preoperative Computed Tomograph (CT) revealed a solid oval hypodense neoformation with regular margins (Dmax 32 mm) without macroscopic adipose tissue located on the anterior side of the left kidney, in its middle portion. This neoformation was characterized by progressive impregnation of contrast and was slightly inhomogeneous, with more hyperdense thin components in the late phase of the dynamic study (Fig. 1). Magnetic Resonance Imaging (MRI) described an expansive neoformation with a solid type signal increasing inhomogeneously after contrast. Diffusion-Weighted Imaging (DWI) findings showed a restriction of Brownian motion of water molecules with suspicious characters in the discariokinetic sense. The lesion was primarily compatible with oncocytoma or “lipid poor” angiomyolipoma, but other primary renal malignant lesions cannot be completely excluded. Abdominal lympho-adenomegalies were not present. The case was discussed in multidisciplinary tumor board and, as there was no reliable data on the benign or malignant nature of the lesion which, in turn, determined the type of surgical resection (total nephrectomy versus partial nephrectomy), it was decided to carry out an ultrasound-guided percutaneous biopsy. After a histological diagnosis of MA, the patient was successively subjected to a left partial nephrectomy. The postoperative course was optimal, and patient was discharged on the fifth postoperative day. Histological examination on partial nephrectomy confirmed the MA diagnosis.

### Pathological findings

Macroscopically the lesion appeared as a capsulated whitish nodule of 3.2 × 3 × 2.5 cm, sometimes with a microcystic appearance. No necrosis or hemorrhagic area was grossly identified.

**Fig. 2:**
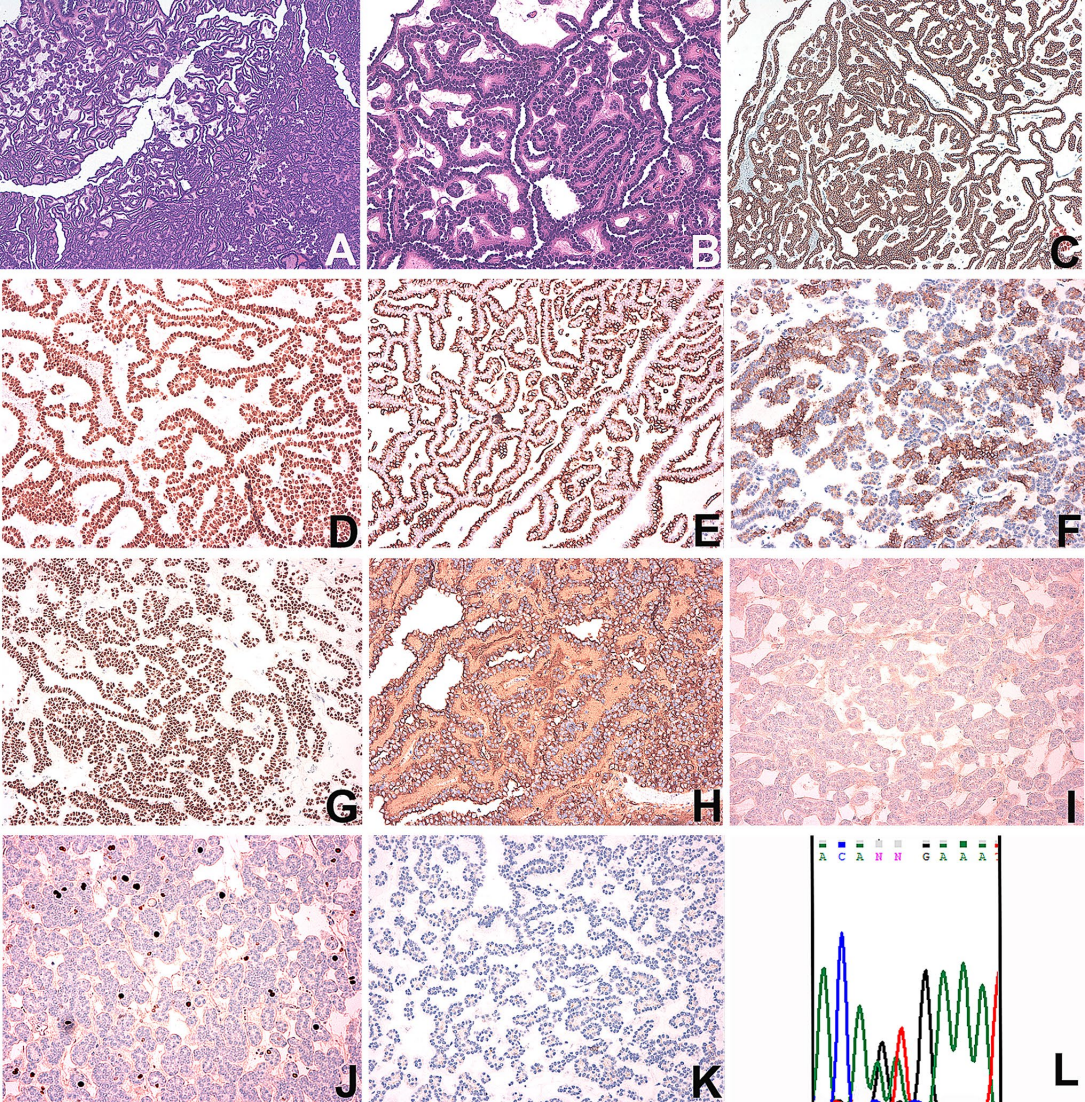
Histological, immunohistochemical and molecular findings. **a** and **b** show the histopathological features of metanephric adenoma with a mix of acinar pattern and papillary structures (E&E; **a** 50 ×  and **b** 200 × magnification). This MA shows a strong positivity for cytokeratins AE1/AE3 (cytoplasmic staining, **c** 200 ×  magnification), PAX8 (nuclear staining, **d** 200 ×  magnification), WT1 (nuclear staining, **g** 200 ×  magnification), CD57 (cytoplasmic staining, **h** 200 ×  magnification), a focal positivity for Vimentin and CK7 (cytoplasmic staining, **e** and **f**, respectively; 200 ×  magnification) and negativity for BRAF VE1 and P504S (**i** and **k**, respectively; 200 ×  magnification). Proliferative index (Ki-67) was around 2–3% (**j** 200 ×  magnification). **l** shows the chromatographic image of heterozygous BRAF V600K mutation (c.1798_1799GT > AA)

Definitive histological examination showed a predominantly acinar neoplasm, sometimes tubular, consisting of neoplastic elements forming papillae covered by an epithelial monolayer, with interposed edematous stroma. Sometimes microcyst formations are present. Neoplastic cells showed regular medium-sized nuclei without evident nucleoli. Psammomatous formations are absent and mitotic figures were rare. Vascular invasion and necrosis were not present and surgical margins were negative (Fig. 2).

Neoplastic elements showed a diffuse positivity for cytokeratins AE1/AE3, CD57, WT1, PAX8, a focal positivity for CK7 and vimentin and negativity for BRAF VE1, CD10, P504S, CK20 (Table1; Fig. 2). Proliferative index (Ki-67) was around 2–3%.

Since the immunophenotypic analysis appeared doubtful, especially for the negativity of the BRAF VE1 antibody, and since MA with mutations other than BRAF V600E have been described, we performed a mutational analysis of the BRAF gene [6]. Metanephric adenoma showed a BRAF V600K mutation (p.Val600Lys; c.1798_1799GT > AA; Fig. 2).

## Discussion

Metanephric adenoma is a rare benign neoplasm that poses two types of problems: the first regarding the preoperative diagnosis, to perform as much as possible a conservative surgical treatment and the second regarding the post-resection histological diagnosis. In fact, MA often enters differential diagnosis with Wilms’ tumor, oncocytoma and papillary renal cell carcinoma [1, 2].

Radiographical images can be useful but nevertheless no reliable imaging findings can definitively distinguish MA from other renal tumors [3], as demonstrated in the present case. In fact, neither the CT nor the MRI imaging with the analysis of the DWI and the apparent diffusion coefficient (ADC) mapping, which proved to be sensitive diagnostic indicators, were conclusive. Total nephrectomy was probably performed in many previously reported cases as MA could not be radiologically distinguished from a malignant tumor. Therefore, when the radiological imaging is not completely clear (malignant versus benign lesion), quite frequent in case of metanephric adenoma, an ultrasound-guided percutaneous biopsy of tumor could be an extremely useful diagnostic approach to diagnose MA and then to perform a conservative surgical treatment.

The histological diagnosis of MA was quite complicated, and this not only for the morphological aspect of the lesion but also for the immunophenotypic pattern. Morphologically, the tumor pattern reminded the papillary renal cell carcinoma type 1 or epithelial-predominant nephroblastoma or oncocytoma. Some morphological elements can help in the differential diagnosis. The presence of psammomatous bodies and foamy macrophages in the axis of the papillae are more typical of the papillary renal cell carcinoma, while a mixture of primitive blastemal cells, epithelial cells and mesenchymal elements and a frequent presence of mitosis is more typical of nephroblastoma and finally the presence of an edematous stroma and oncocyte cells with eosinophilic cytoplasm is instead typical of oncocytoma. However, these distinctive elements may be absent. For example, there may be variants of epithelial-predominant nephroblastoma that closely resemble the metanephric adenoma. The use of immunohistochemistry is frequently useful in the differential diagnosis, being MA cells positive for CD57, WT1 and BRAF VE1 and negative for CK7 and P5014S, but it has given some interpretative problems in this case. Indeed, tumor cells were positive for CD57 and WT1 and negative for P504S but also showed a partial positivity for CK7 and the immunohistochemical analysis for BRAF VE1 antibody was negative. As the high frequency of BRAF V600E mutation in MA, Pinto A. and colleagues has recently postulated that the specific antibody against this alteration (clone VE1) would be valuable diagnostically. Only a mutational analysis demonstrated the presence of the BRAF V600K mutation explaining the negativity for VE1 antibody, able to recognize the BRAF V600E mutation but no other activating BRAF V600 mutations such as the BRAF V600D and the BRAF V600K (described for the first time in this case) [6]. Moreover, Caliò et al. [2] demonstrated that VE1 antibody is a very specific (100%) but less sensitive (80%) marker for identifying BRAF V600E mutated MA.

## Conclusion

In conclusion, the diagnostic approach to metanephric adenoma should be cautious because, in case of an unclear radiographical imaging and to avoid an overtreatment, it may be helpful to perform a percutaneous preoperatory biopsy. Moreover, in the histological diagnosis of MA, the VE1 antibody could be useful but, in case of negativity, a mutational analysis is necessary because of the incomplete sensitivity of the VE1 antibody and the possibility of other activating BRAF mutations than V600E mutation.

## Data Availability

Data and material are available on request.

## Abbreviations

MA: Metanephric adenoma
RCCs: Renal cell carcinomas
CT: Computed tomograph
MRI: Magnetic resonance imaging
DWI: Diffusion-weighted imaging
ADC: Apparent diffusion coefficient
